# Glycemic control is not related to cerebral small vessel disease in neurologically asymptomatic individuals with type 1 diabetes

**DOI:** 10.1007/s00592-021-01821-8

**Published:** 2021-11-15

**Authors:** Jussi Inkeri, Krishna Adeshara, Valma Harjutsalo, Carol Forsblom, Ron Liebkind, Turgut Tatlisumak, Lena M. Thorn, Per-Henrik Groop, Sara Shams, Juha Martola, Jukka Putaala, Daniel Gordin, on behalf of the FinnDiane Study Group

**Affiliations:** 1 HUS Medical Imaging Center, Radiology, University of Helsinki and Helsinki University Hospital, Helsinki, Finland; 2 Folkhälsan Institute of Genetics, Folkhälsan Research Center, Helsinki, Finland; 3 Department of Nephrology, University of Helsinki and Helsinki University Hospital, Helsinki, Finland; 4 Research Program for Clinical and Molecular Metabolism, University of Helsinki, Helsinki, Finland; 5 Neurology, University of Helsinki and Helsinki University Hospital, Helsinki, Finland; 6 Department of Clinical Neuroscience/Neurology, Institute of Neuroscience and Physiology, Sahlgrenska Academy, University of Gothenburg, Gothenburg, Sweden; 7 Department of Neurology, Sahlgrenska University Hospital, Gothenburg, Sweden; 8 Department of General Practice and Primary Health Care, University of Helsinki and Helsinki University Hospital, Helsinki, Finland; 9 Department of Diabetes, Central Clinical School, Monash University, Melbourne, Australia; 10 Department of Radiology, Karolinska University Hospital, Stockholm, Sweden; 11 Department of Clinical Neuroscience, Karolinska Institute, Stockholm, Sweden; 12 Department of Radiology, Stanford University, Stanford, CA USA; 13 Minerva Foundation Institute for Medical Research, Helsinki, Finland; 14 Joslin Diabetes Center, Harvard Medical School, Boston, MA USA

**Keywords:** Cerebral small vessel disease, Magnetic resonance imaging, Fructosamine, Glycated albumin, Long-term glycemic fluctuations

## Abstract

**Aims:**

To determine if medium- and long-term blood glucose control as well as glycemic variability, which are known to be strong predictors of vascular complications, are associated with underlying cerebral small vessel disease (cSVD) in neurologically asymptomatic individuals with type 1 diabetes.

**Methods:**

A total of 189 individuals (47.1% men; median age 40.0, IQR 33.0–45.2 years) with type 1 diabetes (median diabetes duration of 21.7, IQR 18.3–30.7 years) were enrolled in a cross-sectional retrospective study, as part of the Finnish Diabetic Nephropathy (FinnDiane) Study. Glycated hemoglobin (HbA_1c_) values were collected over the course of ten years before the visit including a clinical examination, biochemical sampling, and brain magnetic resonance imaging. Markers of glycemic control, measured during the visit, included HbA_1c_, fructosamine, and glycated albumin.

**Results:**

Signs of cSVD were present in 66 (34.9%) individuals. Medium- and long-term glucose control and glycemic variability did not differ in individuals with signs of cSVD compared to those without. Further, no difference in any of the blood glucose variables and cSVD stratified for cerebral microbleeds (CMBs) or white matter hyperintensities were detected. Neither were numbers of CMBs associated with the studied glucose variables. Additionally, after dividing the studied variables into quartiles, no association with cSVD was observed.

**Conclusions:**

We observed no association between glycemic control and cSVD in neurologically asymptomatic individuals with type 1 diabetes. This finding was unexpected considering the large number of signs of cerebrovascular pathology in these people after two decades of chronic hyperglycemia and warrants further studies searching for underlying factors of cSVD.

**Supplementary Information:**

The online version contains supplementary material available at 10.1007/s00592-021-01821-8.

## Introduction

High blood glucose is a major risk factor for not only microvascular complications, but also cardiovascular disease in type 1 diabetes [1, 2]. Cardiovascular complications cause significant premature mortality in individuals with type 1 diabetes [3]. Despite the fact that type 1 diabetes increases the risk of stroke fourfold compared to non-diabetic individuals, this grim complication has been less studied than other cardiovascular consequences [4]. We observed recently that a third of neurologically asymptomatic individuals with type 1 diabetes showed signs of pathological cerebral small vessel disease (cSVD), however, virtually none among the healthy control subjects. Of the different manifestations, white matter hyperintensities (WMHs) were observed in 17% and cerebral microbleeds (CMBs) in 24% in our cohort comprised of individuals with type 1 diabetes and a mean age of 40.0 [5]. Our findings resemble those of the Pittsburgh EDC study reporting 33% of individuals with a mean age of 49.5 years showing signs of white matter hyperintensities (WMHs) in brain magnetic resonance imaging (MRI) [6]. As hemosiderin-sensitive sequences were not part of the MRI protocol in the Pittsburgh cohort CMBs could not be detected.

Notably, only few of the traditional risk factors were different in type 1 diabetes individuals with and without cSVDs. Blood pressure, a well-known risk factor for cSVD [7], was higher in both individuals with WMHs and CMBs compared to those without [5], and especially nocturnal hypertension was associated cSVD [8]. However, it is unlikely that the modestly higher blood pressure in individuals with cSVD compared to those with no cerebrovascular pathology would fully explain this finding [5]. Neither could we observe a difference in HbA_1c_ at the time of the imaging study. This warrants further analysis of glycemic control in relation to cSVD in this type 1 diabetes cohort with more than two decades of hyperglycemia.

The aim of this study was to retrospectively determine whether medium- or long-term blood glucose control measured by different markers were associated with cSVD in neurologically asymptomatic individuals with type 1 diabetes. Additionally, we sought to investigate whether long-term glycemic fluctuations, known to predict vascular complications in this patient group, are predictive of cSVD.

## Methods

This study was performed as part of the Finnish Diabetic Nephropathy (FinnDiane) Study, a nationwide multicenter study aiming to identify genetic, environmental, and clinical risk factors for micro- and macrovascular complications in type 1 diabetes [5]. A total of 191 individuals with type 1 diabetes were enrolled to the study. Two individuals were excluded due to missing clinical data. Thus, a total of 189 individuals with type 1 diabetes were included in the present study. Age span ranged between 18 and 50 years and the onset of diabetes was < 40 years. Individuals with renal replacement therapy, any clinical signs of cerebrovascular disease, or contraindications for MRI were excluded from this substudy. The study was carried out in accordance with the Declaration of Helsinki and approved by the Ethics Committee of the Helsinki and Uusimaa Hospital District. Each participant signed a written informed consent [5].

All individuals were studied at the FinnDiane Research Center (Biomedicum) and the Medical Imaging Center at Helsinki University Hospital, both in Helsinki, Finland. Clinical visits included brain MRI scans, biochemical sampling, and a thorough clinical examination. The study visits and methods have been presented in greater detail before [5]. Briefly, brain MRI was performed with a 3.0 T scanner (Achieva; Philips, Best, the Netherlands). The images were assessed by an experienced neuroradiologist (JM) who was blinded to all clinical data. Markers of cSVD were rated per the standardized STRIVE criteria, including the assessment of WMHs (Fazekas scale used, with category ≥ 1 considered a significant burden), CMBs, and lacunar infarcts [9].

### Measures of blood glucose control

To characterize medium-term glucose control, fructosamine (FA), and glycated albumin (GA), reflecting blood glucose during a time span of two to three weeks, were measured [10, 11]. Blood glycated hemoglobin (HbA_1c_), reflecting blood glucose control during a time span of one to two months, was measured using standardized assays in a central laboratory (Medix Laboratories, Espoo Finland) [12]. Three or more HbA_1c_ values over the course of ten years before the visit (median count 16, IQR 10–23) were obtained in order to calculate overall mean HbA_1c_ (HbA_1c_-mean_overall_) for each individual to better delineate long-term glucose control. These values were collected from local laboratories using standardized methods (HPLC) with a normal range of 4–6%. Measurements of HbA_1c_ visit-to-visit variability reflects long-term blood glucose fluctuations in a wider timespan of months to years [13]. To assess long-term blood glucose fluctuations HbA_1c_ standard deviation (HbA_1c_-SD), HbA_1c_ coefficient of variation (HbA_1c_-CV), and HbA_1c_ average real variability (HbA_1c_-ARV) were calculated for each individual. To minimize any effect of a varying number of HbA_1c_ values on long-term glucose variability, adjusted HbA_1c_ standard deviation (HbA_1c_-adjSD) were defined for each individual. Of the individuals with type 1 diabetes, 44 had less than three HbA_1c_ values available ten years before the visit**,** three had missing data on FA or GA and were excluded from the respective analyses.

### Determination of glycated albumin (GA)

GA concentration was determined according to manufacturers' instructions using a competitive ELISA kit (Human glycated albumin ELISA Kit, CSB-E09599h, Cusabio, Wuhan, Hubei Province, China) [14]. Samples were diluted to 1:250 with the sample diluent buffer provided with the kit to achieve sample absorbance within the range of a standard curve. The absorbance was measured at 450 nm using a Synergy H1 hybrid multi-mode microplate reader (Biotek, Winooski, VT, USA). The amount of GA was determined by comparing with the known standard provided with the kit and expressed as nM/ml of GA present in human serum samples.

### Determination of fructosamine (FA)

Serum FA levels were measured by colorimetric technique based on the ability of FA to reduce nitroblue tetrazolium (NBT) to tetrazinolyl radical NBT + , which further yields formation of colored formazan under alkaline condition [15]. The developed color intensity was measured at 540 nm and FA content was calculated using standard 1-deoxy-1 morpholino-D-fructose (0–3.2 mM/L).

### Statistics

Statistical analyses were performed using IBM SPSS Statistics 26.0 (IBM, Armonk, NY). *T*-tests were used for parametric data and presented as means (± SD), and Mann–Whitney-*U* or Kruskal–Wallis tests for the nonparametric data presented as medians (interquartile range). The *X*^2^ test or Fisher’s exact tests were performed for categorical variables. HbA_1c_-adjSD was calculated according to the formula: $\mathrm{SD}/\sqrt{[\mathrm{n}/(\mathrm{n}-1)]}$ [16, 17]. HbA_1c_-CV was calculated as the HbA_1c_ (%) SD divided by the mean and multiplied by 100, result presented as a percentage and HbA_1c_-ARV as the average of the absolute differences between consecutive HbA_1c_ (%) measurements [18]. The study individuals were divided into three groups based on the number of CMBs (zero, one to two, more than two) and into quartiles based on the HbA_1c_, FA, GA, HbA_1c_-mean_overall_, HbA_1c_-SD, HbA_1c_-adjSD, HbA_1c_-CV, and HbA_1c_-ARV values. Bivariate (Pearson) correlation analysis was used to study correlations between HbA_1c_, FA, GA, and HbA_1c_-mean_overall_. The threshold for statistical significance was set at *p* < 0.05.

## Results

### Clinical characteristics

**Fig. 1:**
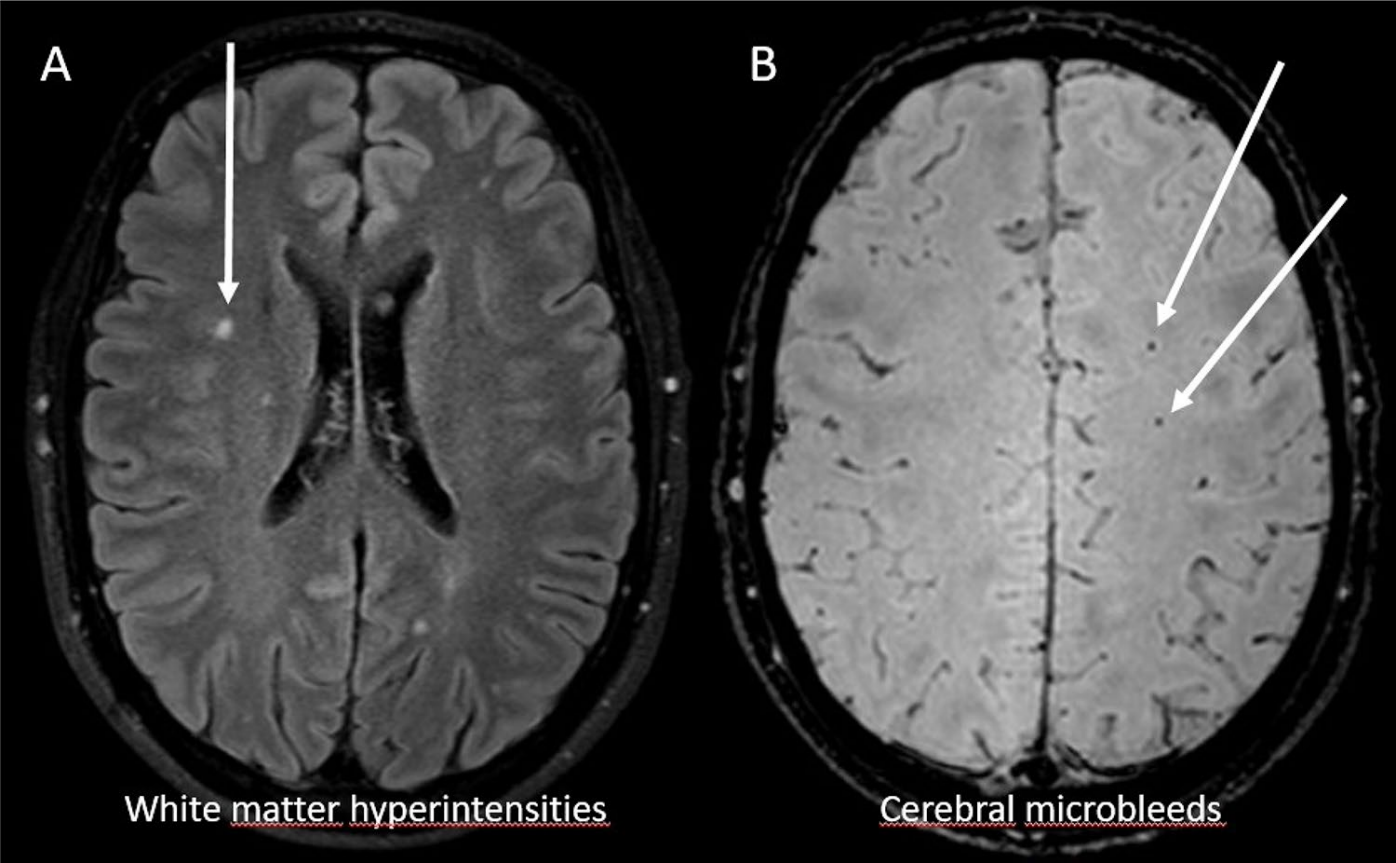
MRI findings of cerebral small vessel disease. Fluid attenuated inversion recovery image (FLAIR) with white matter hyperintensity (arrow) (**a**). Susceptibility weighted image (SWI) with cerebral microbleeds (arrows) (**b**)

One hundred and eighty-nine individuals with type 1 diabetes were enrolled for this study, with demographics previously presented in greater detail [5]. Briefly, the median age of the individuals with type 1 diabetes was 40.0 (33.0–45.2) years, 47.1% were male and median diabetes duration was 21.7 (18.3–30.7) years. One individual had a history of an acute myocardial infarction, no other cardiovascular events were recorded. Mean systolic blood pressure was 130 ± 14 mmHg. Among cases, 31 (16.9%) had albuminuria, 20 (10.9%) microalbuminuria, and 11 (6.0%) macroalbuminuria. Sixty-six (34.9%) showed signs of cSVD, 45 (23.8%) had CMBs, 32 (16.9%) WMHs, and 4 (2.1%) lacunar infarcts. The overlap between these changes was eleven (5.8%) for CMBs and WMHs and two (1.1%) for both CMBs or WMHs and lacunar infarct. Examples of these MRI findings are presented in Fig. 1. Fifty-five (29.1%) of the individuals were on insulin pump treatment. Insulin pump treatment did not correlate with the presence of cSVD (data not shown). Median HbA_1c_, GA, and FA values during the visits were 8.1% (7.4–8.9%), (65.0 mmol/mol [57.0–73.0 mmol/mol]), 91.6 nM/ml (74.3–116.4 nM/ml), and 2.6 mM/l (2.4–3.0 mM/l), respectively. HbA_1c_-mean_overall_, collected over the course of ten years before the visit (median count 16, IQR 10–23), were 8.1 ± 0.9% (65.4 ± 10.3 mmol/mol) (Table 1). Bivariate correlations between HbA_1c_, FA, GA, and HbA_1c_-mean_overall_ are presented in Supplementary Table 1. An association was observed between HbA_1c_ vs. FA (*p* = 0.018) and HbA_1c_ vs. HbA_1c_-mean_overall_ (*p* < 0.001). To overcome the possibility of bias by the number of HbA_1c_ measurements we divided the study individuals into two groups, above and below median HbA_1c_ count. The presence of cSVD were not different between the groups (24 [30.8%] vs. 29 [43.3%], *p* = 0.119).

**Table 1:** Clinical characteristics of the study population

	Individuals with type 1 diabetes *n* = 189
Age, years	40.0 (33.0–45.2)
Male sex	89 (47.1)
Diabetes duration, years	21.7 (18.3–30.7)
Cerebral small vessel disease	66 (34.9)
Cerebral microbleeds	45 (23.8)
White matter hyperintensities	32 (16.9)
Lacunae	4 (2.1)
Systolic blood pressure, mmHg	130 ± 14
Total cholesterol, mmol/L, median	4.4 (4.0–4.9)
High-density lipoprotein, mmol/L, median	1.4 (1.2–1.7)
Low-density lipoprotein, mmol/L, median	2.4 (2.1–3.0)
Triglycerides, mmol/L, median	0.9 (0.7–1.4)
Albuminuria	31 (16.9)
Microalbuminuria	20 (10.9)
Macroalbuminuria	11 (6.0)
Estimated glomerular filtration rate, ml/min/1.73 m^2^	108.2 (96.4–115.8)
HbA_1c_, %	8.1 (7.4–8.9)
HbA_1c_, mmol/mol	65.0 (57.0–73.0)
Glycated albumin, nM/ml	91.6 (74.3–116.4)
Fructosamine, mM/l	2.6 (2.4–3.0)
HbA_1c_ count, n	16 (10–23)
HbA_1c_-mean_overall,_ %	8.1 ± 0.9
HbA_1c_-mean_overall,_ mmol/mol	65.4 ± 10.3
HbA_1c_ standard deviation, %	0.59 (0.44–0.81)
HbA_1c_ adjusted standard deviation, %	0.56 (0.43–0.76)
HbA_1c_ coefficient of variation, %	7.2 (5.6–9.6)
HbA_1c_ average real variability	0.5 (0.4–0.6)

Data are *n* (%), median (interquartile range) or mean ± SD unless otherwise indicated

Individuals with CMBs or WMHs had higher systolic blood pressure compared to those without CMBs or WMHs (135 $\pm$ 17 mmHg vs. 129 $\pm$ 13 mmHg, *p* = 0.011 for CMBs and 137 $\pm$ 15 mmHg vs. 129 $\pm$ 14 mmHg, *p* = 0.005 for WMHs). The presence of WMHs correlated also with age (45.0 [40.4–47.6] years vs. 38.6 [32.5–44.2] years, *p* < 0.001) and the presence of CMBs with albuminuria (13 [30.2%] vs. 18 [12.9%], *p* = 0.008). The other demographic variables were not associated with CMBs or WMHs.

### Medium- and long-term blood glucose control and cSVD

HbA_1c_ at the study visit did not correlate with the presence of cSVD (8.2% [7.6–8.9%], 66.0 mmol/mol [59.8–73.3 mmol/mol] vs. 8.0% [7.3–8.8%], 64.0 mmol/mol [56.0–73.0 mmol/mol], *p* = 0.259), CMBs, or WMHs in individuals with type 1 diabetes. GA and FA did not correlate with cSVD (97.2 [73.9–117.8] nM/ml vs. 89.6 [76.3–115.9] nM/ml, *p* = 0.704 for GA and 2.6 [2.4–2.9] mM/l vs. 2.5 [2.3–3.0] mM/l *p* = 0.587 for FA), CMBs, or WMHs in brain MRIs (Table 2). Furthermore, individuals with type 1 diabetes divided into quartiles based on their HbA_1c_, GA, and FA values showed no correlations with the presence on cSVD markers (Table 3). Neither did we observe associations between HbA_1c_, GA, and FA and the number of CMBs (Table 4).

**Table 2:** HbA_1c_, glycated albumin, fructosamine, and long-term glycemic variability stratified by small vessel disease findings in brain MRI in individuals with type 1 diabetes

	Cerebral small vessel disease			Cerebral microbleeds			White matter hyperintensities		
	Presence (*n* = 66)	Absence (*n* = 123)	*p*	Presence (*n* = 45)	Absence (*n* = 144)	*p*	Presence (*n* = 32)	Absence (*n* = 157)	*p*
HbA_1c_, %, (mmol/mol)	8.2 (7.6–8.9), (66.0 [59.8–73.3])	8.0 (7.3–8.8), (64.0 [56.0–73.0])	0.259	8.2 (7.6–8.9), (66.0 [60.0–73.0])	8.1 (7.4–8.8), (65.0 [57.0–73.0])	0.419	8.2 (7.4–8.7), (65.5 [58.3–72.0])	8.1 (7.4–8.9), (65.0 [57.0–73.0])	0.838
GA, nM/ml	97.2 (73.9–117.8)	89.6 (76.3–115.9)	0.704	102.9 (73.9–125.3)	90.3 (76.3–115.2)	0.580	98.0 (82.6–113.7)	89.8 (74.0–117.8)	0.492
FA, mM/l	2.6 (2.4–2.9)	2.5 (2.3–3.0)	0.587	2.6 (2.4–2.9)	2.5 (2.3–3.0)	0.439	2.6 (2.4–3.0)	2.6 (2.4–3.0)	0.429
HbA_1c_-mean_overall_, %, (mmol/mol)	8.3 ± 1.0, (67.4 ± 11.2)	8.0 ± 0.9, (64.2 ± 9.5)	0.141	8.3 ± 0.9, (67.2 ± 10.3)	8.1 ± 0.9, (64.7 ± 10.2)	0.280	8.2 ± 1.1, (66.4 ± 12.0)	8.1 ± 0.9, (65.1 ± 9.9)	0.787
HbA_1c_-SD, %	0.57 (0.42–0.78)	0.61 (0.44–0.81)	0.655	0.56 (0.42–0.76)	0.61 (0.44–0.82)	0.514	0.52 (0.39–0.97)	0.61 (0.46–0.81)	0.445
HbA_1c_-adjSD, %	0.55 (0.40–0.73)	0.58 (0.43–0.78)	0.771	0.53 (0.40–0.72)	0.58 (0.43–0.79)	0.577	0.51 (0.39–0.93)	0.57 (0.44–0.75)	0.480
HbA_1c_-CV, %	6.7 (5.5–8.7)	7.6 (5.7–9.9)	0.245	6.7 (5.5–8.5)	7.4 (5.7–10.1)	0.219	6.0 (5.0–11.5)	7.5 (5.8–9.5)	0.293
HbA_1c_-ARV	0.5 (0.4–0.6)	0.5 (0.3–0.7)	0.953	0.5 (0.4–0.6)	0.5 (0.4–0.6)	0.578	0.4 (0.4–0.6)	0.5 (0.4–0.7)	0.410

Data are median (interquartile range) or mean ± SD unless otherwise indicated. GA = glycated albumin, FA = fructosamine, SD = standard deviation, adjSD = adjusted standard deviation, CV = coefficient of variation, ARV = average real variability

**Table 3:** HbA_1c_, glycated albumin, fructosamine, and long-term glycemic variability lowest and highest quartile crosstabs by small vessel disease findings in brain MRI in individuals with type 1 diabetes

	Cerebral small vessel disease			Cerebral microbleeds			White matter hyperintensities		
	Presence	Absence	*p*	Presence	Absence	*p*	Presence	Absence	*p*
HbA_1c_, highest quartile	18 (54.4)	29 (56.7)	0.289	12 (57.1)	35 (44.3)	0.295	7 (46.7)	40 (47.1)	0.978
HbA_1c_, lowest quartile	15 (45.5)	38 (56.7)		9 (42.9)	44 (55.7)		8 (53.3)	45 (52.9)	
GA, highest quartile	17 (50)	29 (50)	1.000	14 (53.8)	32 (48.5)	0.643	6 (54.5)	40 (49.4)	0.748
GA, lowest quartile	17 (50)	29 (50)		12 (46.2)	34 (51.5)		5 (45.5)	41 (50.6)	
FA, highest quartile	14 (53.8)	33 (50.8)	0.791	10 (58.8)	37 (50)	0.512	10 (62.5)	37 (49.3)	0.339
FA, lowest quartile	12 (46.2)	32 (49.2)		7 (41.2)	37 (50)		6 (37.5)	38 (50.7)	
HbA_1c_-mean_overall_, highest quartile	16 (57.1)	20 (45.5)	0.334	11 (57.9)	25 (47.2)	0.422	7 (50)	29 (50)	1.000
HbA_1c_-mean_overall_, lowest quartile	12 (42.9)	24 (54.5)		8 (42.1)	28 (52.8)		7 (50)	29 (50)	
HbA_1c_-SD, highest quartile	12 (48.0)	24 (52.2)	0.737	7 (43.8)	29 (52.7)	0.527	7 (46.7)	29 (51.8)	0.725
HbA_1c_-SD, lowest quartile	13 (52.0)	22 (47.8)		9 (56.3)	26 (47.3)		8 (53.3)	27 (48.2)	
HbA_1c_-adjSD, highest quartile	12 (44.4)	24 (51.1)	0.583	7 (38.9)	29 (51.8)	0.341	7 (41.2)	29 (50.9)	0.482
HbA_1c_-adjSD, lowest quartile	15 (55.6)	23 (48.9)		11 (61.1)	27 (48.2)		10 (58.8)	28 (49.1)	
HbA_1c_-CV, highest quartile	12 (44.4)	24 (53.3)	0.465	7 (41.2)	29 (52.7)	0.405	7 (41.2)	29 (52.7)	0.405
HbA_1c_-CV, lowest quartile	15 (55.6)	21 (46.7)		10 (58.8)	26 (47.3)		10 (58.8)	26 (47.3)	
HbA_1c_-ARV, highest quartile	11 (54.2)	25 (48.1)	0.739	9 (56.3)	27 (47.4)	0.530	4 (44.4)	32 (50)	0.518
HbA_1c_-ARV, lowest quartile	10 (47.6)	27 (51.9)		7 (43.8)	30 (52.6)		5 (55.6)	32 (50)	

Data are n (%). GA = glycated albumin, FA = fructosamine, SD = standard deviation, adjSD = adjusted standard deviation, CV = coefficient of variation, ARV = average real variability

**Table 4:** HbA_1c_, glycated albumin, fructosamine and long-term glycemic variability by number of cerebral microbleeds in individuals with type 1 diabetes

	Number of cerebral microbleeds			
	0 (*n* = 144)	1–2 (*n* = 33)	3 or more (*n* = 12)	*p*
HbA_1c_, %, (mmol/mol)	8.1 (7.4–8.8), (65.0 [57.0–73.0])	8.2 (7.6–8.8), (66.0 [59.5–72.0])	8.3 (7.7–9.4), (66.5 [61.3–79.0])	0.470
GA, mM/l	90.3 (76.3–115.2)	98.4 (72.4–121.9)	106.9 (81.8–133.0)	0.677
FA, mM/l	2.5 (2.3–3.0)	2.5 (2.4–2.9)	2.8 (2.8–3.1)	0.066
HbA_1c_-mean_overall_, %, (mmol/mol)	8.1 ± 0.9, (64.7 ± 10.2)	8.2 ± 0.8, (66.2 ± 8.8)	8.6 ± 1.2, (70.1 ± 13.6)	0.403
HbA_1c_-SD, %	0.61 (0.44–0.82)	0.57 (0.39–0.83)	0.55 (0.48–0.68)	0.702
HbA_1c_-adjSD, %	0.58 (0.42–0.79)	0.56 (0.36–0.76)	0.52 (0.45–0.66)	0.735
HbA_1c_-CV, %	7.4 (5.7–10.1)	6.8 (5.4–9.8)	6.6 (5.5–7.9)	0.334
HbA_1c_-ARV	0.5 (0.4–0.6)	0.5 (0.4–0.6)	0.6 (0.4–0.7)	0.718

Data are median (interquartile range) or mean ± SD unless otherwise indicated. GA = glycated albumin, FA = fructosamine, SD = standard deviation, adjSD = adjusted standard deviation, CV = coefficient of variation, ARV = average real variability

Differences in HbA_1c_-mean_overall_ value, collected within ten years prior to the study visit, were not observed between those type 1 diabetes individuals with any signs of cSVD in their brain MRIs compared to those without (8.3 ± 1.0% [67.4 ± 11.2 mmol/mol] vs. 8.0 ± 0.9% [64.2 ± 9.5 mmol/mol], *p* = 0.141) (Table 2). This was also true when analyzing separately the cerebral changes CMBs and WMHs. We observed no associations between cumulative blood glucose values and cSVDs or the number of CMBs after dividing individuals with type 1 diabetes into quartiles based on the HbA_1c_-mean_overall_ (Tables 3 and 4).

### Glycemic variability and cSVD

Long-term HbA_1c_ variability, measured as HbA_1c_-SD (0.57% [0.42–0.78%] vs. 0.61% [0.44–0.81%], *p* = 0.655, HbA_1c_-adjSD (0.55% [0.40–0.73%] vs. 0.58% [0.43–0.78%], *p* = 0.771, HbA_1c_-CV (6.7% [5.5–8.7%] vs. 7.6% [5.7–9.9%], *p* = 0.245), and HbA_1c_-ARV (0.5 [0.4–0.6] vs. 0.5 [0.3–0.7], *p* = 0.953), did not correlate with the presence of cSVD. Similarly, no correlation was observed between glycemic variability and WMHs, CMBs, or the number of CMBs (Tables 2 and 4). After dividing the population into quartiles of HbA_1c_ variability, no correlation was observed with the presence of cSVD, CMBs or WMHs observed in brain MRI (Table 3).

## Discussion

The main finding of our study was that medium- and long-term blood glucose control and glycemic variability showed no association with cSVD in neurologically asymptomatic individuals with type 1 diabetes after two decades of chronic hyperglycemia. Our study results suggest that factors other than blood glucose control are central in the development of cSVD in type 1 diabetes.

Risk factors for cSVD, especially for CMBs, are scarcely studied in type 1 diabetes. The Pittsburgh EDC study reported no association between WMHs and chronic hyperglycemia measured as HbA_1c_ [6]. Similar findings were reported in another cohort consisting of 114 individuals with type 1 diabetes [19]. Our findings are in concordance with these previous studies, further extending their observations by carefully characterizing blood glucose control as well as deepening the cerebrovascular phenotype. We measured cumulative blood glucose and glycemic variability after collecting HbA_1c_ values over a course of ten years before the study visit. Furthermore, medium-term glucose control was estimated by adding two established glycemic markers, namely FA and GA, into the analyses. Lastly, in contrast to prior studies, CMBs being strongly associated with future strokes and mortality [20, 21] were identified from brain MRI scans in our study in contrast to only WMHs and lacunes in previous studies.

A third of the individuals in our population of neurologically asymptomatic individuals with type 1 diabetes showed signs of pathological cSVD. However, hardly any cerebrovascular changes were observed in the normoglycemic healthy control subjects. Only a few established clinical risk factors were different in individuals with and without cSVDs. Notably, differences in these risk factors, namely blood pressure and albuminuria, were only modestly explaining the cerebral findings [5]. It is, thus, surprising that variables reflecting blood glucose control at the time of the brain MRI study, cumulative blood glucose levels prior to the study, or blood glucose variability showed no associations with vascular pathology detected in brain MRI.

Individuals with type 1 diabetes have a markedly increased risk for cardiovascular morbidity and mortality compared to the healthy population [22]. We have previously shown that HbA_1c_ is an independent risk factor for ischemic but not for hemorrhagic stroke [23]. Similarly, intensive diabetes therapy reduced a pooled cardiovascular disease (CVD) end-point consisting of nonfatal myocardial infarction, stroke and death by 57 percent in the Diabetes Control and Complications Trial (DCCT) and the Epidemiology of Diabetes Interventions and Complications (EDIC) Study [2]. It may well be that CVD outcomes in these longitudinal studies were partly secondary to diabetic kidney disease (DKD), a strong risk factor for cerebrovascular disease, whereas 83.1% of the participants in our study, showed no signs of DKD. This raises the question whether the detrimental effect of hyperglycemia on the cerebrovascular bed is mediated via diabetic microvascular complications, and kidney disease in particular.

Glycemic variability has been suggested to cause cellular damage in different organs, particularly via oxidative stress [24]. We have shown long-term glucose variability, measured as SD of longitudinal HbA_1c_ values, to predict incident of microalbuminuria, progression of renal disease, and cardiovascular disease events in type 1 diabetes [13]. Similar findings were reported in another study, where HbA_1c_ variability predicted retinopathy, nephropathy, and cardiac autonomic neuropathy in adolescents with type 1 diabetes [25]. The DCCT Study reported HbA_1c_ variability to contribute to the development of retinopathy and nephropathy, whereas short-term glucose variability did not predict the development of these complications [16, 26, 27]. Previous reports showed no strong association of FA with severity of hemiparesis and predicted stroke outcome in general population with brain infarction of the carotid territory [28] and in individuals with cerebral hemorrhage at an early stage of their illness [29]. Also, GA has shown different impact on stroke outcomes being associated with only large artery atherosclerosis but not with small vessel occlusion and cardioembolism in diabetic individuals with acute ischemic stroke [30]. However, other study reported association of GA with early neurological deterioration in prediabetic individuals with acute ischemic stroke [31]. Reflecting short-term glycemia, FA and GA levels can be affected by acute blood glucose change, albumin turnover or metabolism [32] and therefore reflects its variability in a disease specific manner. These observations and present findings suggest that an abnormal level of glycemic biomarkers reflect metabolic illness but does not exacerbate an acute manifestation of cerebrovascular changes. Future studies are needed to investigate whether short-term glucose control and variability contribute to the risk of cSVD, especially CMBs in type 1 diabetes.

High blood glucose is the main driver of diabetic retinopathy, another form of cerebrovascular disease, in type 1 diabetes [33]. It is thus of interest that the number of CMBs has earlier been shown to be higher in individuals with type 1 diabetes and severe diabetic retinopathy [34]. Similarly, the prevalence of WMHs and/or lacunes has been shown to correlate with diabetic retinopathy in type 2 diabetes [35]. We did also observe an association between CMBs and diabetic retinal disease [36]. This association was, however, independent of HbA_1c_ reflecting the strong relationship between blood glucose and diabetic retinal disease. The findings that the blood glucose levels were associated with diabetic retinopathy albeit not cSVD raises the question, whether the mechanisms of the adverse effects of hyperglycemia on the central nervous system could be different from those in the retina. It may well be that changes in multiple metabolic factors induced by diabetes contribute differently to the abnormalities in the cerebral and the retinal vasculature. Further studies on potential metabolic changes in our cohort are now ongoing to address this question.

It is of note that the glucose levels on both sides of the blood brain barrier, namely blood and cerebrospinal fluid, may not be identical. Important regulators are involved in this delicate balance such as glucose transporters (GLUTs) to maintain the continuous high glucose and energy demands of the brain [37, 38]. Mechanistic studies are warranted to give an answer whether GLUTs could explain these findings. Interestingly, poorly controlled diabetes mellitus can cause a variety of adverse effects on brain function and metabolism via both low and high blood glucose levels [37]. These blood glucose alterations in diabetes mellitus can affect cerebral neurotransmitter metabolism, cerebral blood flow, and blood–brain barrier [37, 39]. Particularly dysfunction of the blood–brain barrier has been suggested to relate to intracerebral hemorrhage and the presence of CMBs [40]. Whether a damaged blood–brain barrier explains the number of CMBs in our cohort is not known. Neither if such changes could be caused by a poor glycemic control.

Our study does not go without limitations. We had serial A1c values from ten years enabling us to assess both cumulative blood glucose control and blood glucose variability. The cross-sectional retrospective nature of the study should, however, be taken into account. Our study had no data regarding short-term glucose control such as time in range (TIR) or variability measured from continuous glucose monitoring systems (CGMS), leaving this interesting topic open for future studies. The number of participants and HbA_1c_ measurements, reflecting long-term blood glucose levels and fluctuations, is limited and this may have an effect on the statistical power to detect differences between the groups. A larger cohort would have enabled greater statistical power. It is, however, improbable that this would markedly have changed the results considering the consistence of the observations. The strengths of this study are the standardized imaging and clinical assessment, as well as the strong phenotypic data.

## Conclusion

We observed no association between medium- and long-term blood glucose control and long-term glycemic variability and cSVD in neurologically asymptomatic individuals with type 1 diabetes. This finding was unexpected considering the large number of signs of cerebrovascular pathology in these people after two decades of chronic hyperglycemia and warrants further studies searching for underlying factors of cSVD.

## Supplementary Information

Below is the link to the electronic supplementary material.

Supplementary file1 (DOCX 21 KB)

## Data Availability

The database is available for all FinnDiane researchers.
