# Supplementary material for: Glycemic control is not related to cerebral small vessel disease in neurologically asymptomatic individuals with type 1 diabetes
Source: Acta Diabetol. 2021 Nov 15;59(4):481–90. doi: 10.1007/s00592-021-01821-8 (PMC8917104; doi:10.1007/s00592-021-01821-8)
Supplement: Supplementary file 1 — Supplementary file1 (DOCX 21 KB) [file 592_2021_1821_MOESM1_ESM.docx]

**Supplementary Table 1** Bivariate correlations between HbA_1c_, glycated albumin, fructosamine and HbA_1c_-mean_overall_ in individuals with type 1 diabetes

|  | **GA** | **FA** | **HbA_1c_-mean_overall_** |
| --- | --- | --- | --- |
| **HbA_1c_** | *R* = 0.029;  *p* = 0.693 | *R* = 0.173;  *p* = 0.018 | *R* = 0.658;  *p* < 0.001 |
| **GA** |  | *R* = -0.103;  *p* = 0.164 | *R* = 0.063;  *p* = 0.459 |
| **FA** |  |  | *R* = 0.158;  *p* = 0.061 |

GA = glycated albumin, FA = fructosamine
